# The Frequency and Association of Dyskalemias With Types of Arrhythmias and Their Predictors in Emergency Cardiac Care Patients

**DOI:** 10.7759/cureus.96528

**Published:** 2025-11-10

**Authors:** Bushra Ghulam Nabi, Muhammad Usama, Qazi M Tufail, Humaira Sami Ullah, Aleen Mushtaq, Syed Fakhar Haider Bukhari, Usama Tariq

**Affiliations:** 1 Surgery, Benenden Hospital, Benenden, GBR; 2 Cardiology, Allama Iqbal Children Hospital, Sialkot, PAK; 3 Cardiology, Shaikh Zayed Hospital, Lahore, PAK; 4 Medicine, Bolan Medical Complex Hospital, Quetta, PAK; 5 Medicine, Saadat Medical Complex, Narowal, PAK; 6 Acute Medicine, Sandwell and West Birmingham Hospitals, Birmingham, GBR

**Keywords:** arrhythmias, cardiac arrhythmia, dyskalemias, emergency, value in health care

## Abstract

Background: Serum potassium is a critical determinant of myocardial excitability and conduction. Dyskalemias can trigger life-threatening arrhythmias, including ventricular tachycardia (VT) and ventricular fibrillation (VF). Even mild derangements in potassium levels can destabilize cardiac electrophysiology and precipitate serious arrhythmias. In emergency cardiac care, dyskalemias are frequently encountered but often underrecognized contributors to electrical instability, morbidity, and mortality.

Objective: To determine the frequency of serum potassium derangements and assess their association with the type of arrhythmias in patients presenting to emergency cardiac care. Thirdly, to determine the co-morbidities that can be predictors of arrhythmias.

Methods: This cross-sectional analytical study was conducted in the Department of Emergency Cardiac Care at Shaikh Zayed Hospital, Lahore, Pakistan, from July 2024 till December 2024, including 185 patients presenting with acute cardiac complaints. Serum potassium levels were measured at admission and categorized as hypokalemia (<3.5 mmol/L), normokalemia (3.5-5.0 mmol/L), or hyperkalemia (>5.0 mmol/L). Electrocardiograms and continuous monitoring were used to document arrhythmic events.

Results: The mean age of patients was 58.6 ± 12.4 years; 60.5% were male. Dyskalemias were present in 50.3% of patients. Hypokalemia was observed in 31.4%, hyperkalemia in 18.9%, and normokalemia in 49.7%. Arrhythmias occurred in 42.7% of cases, with ventricular arrhythmias significantly associated with hypokalemia (36.2% vs. 10.9%, p<0.001), and bradyarrhythmias/conduction blocks linked to hyperkalemia (25.7%, p=0.002). Logistic regression showed hypokalemia independently increased the odds of arrhythmias (OR=4.89; 95% CI: 2.45-9.75; p<0.001). Chronic kidney disease (CKD) was also an independent predictor of overall arrhythmic occurrence (OR=2.11; 95% CI: 1.04-4.27; p=0.03), likely reflecting its dual contribution to both hyperkalemia and electrical instability.

Conclusion: Potassium derangements are common in emergency cardiac patients and significantly increase the risk of arrhythmias. Hypokalemia predisposes to ventricular arrhythmias, while hyperkalemia more often results in bradyarrhythmias and conduction blocks. Chronic kidney disease is an independent predictor of arrhythmias.

## Introduction

Serum potassium is the most abundant intracellular cation, essential for maintaining cellular homeostasis and regulating neuromuscular and cardiac function. In the myocardium, potassium plays a central role in establishing the resting membrane potential and orchestrating repolarization during each action potential [1]. These processes are critical for synchronized myocardial contraction and effective cardiac output. Even small fluctuations in extracellular potassium concentration can significantly influence myocardial excitability, conduction velocity, and refractoriness, thereby predisposing to arrhythmias that can range from asymptomatic premature beats to malignant ventricular arrhythmias or sudden cardiac death [2]. Electrolyte derangements, particularly potassium abnormalities, are among the most frequent metabolic disturbances encountered in emergency cardiac care [3]. Hypokalemia, defined as serum potassium less than 3.5 mmol/L, and hyperkalemia, defined as serum potassium greater than 5.0 mmol/L, are both potentially lethal if not recognized and corrected promptly [4].

Hypokalemia is often precipitated by diuretic therapy, vomiting, diarrhea, malnutrition, or endocrinological disorders such as hyperaldosteronism. Its clinical relevance lies in its ability to delay ventricular repolarization, thereby prolonging the QT interval and creating a substrate for torsades de pointes and ventricular tachyarrhythmias [5]. Hyperkalemia, conversely, arises most commonly in patients with chronic kidney disease, metabolic acidosis, or those receiving potassium-sparing diuretics, angiotensin-converting enzyme (ACE) inhibitors, or angiotensin II receptor blockers (ARBs). Elevated serum potassium decreases the myocardial resting potential, leading to impaired depolarization, conduction delays, and in severe cases, sine-wave electrocardiographic patterns followed by asystole [6]. The burden of arrhythmias in emergency cardiac care is substantial. Arrhythmias not only complicate acute coronary syndromes, heart failure, and structural heart disease, but they may also serve as the primary presentation of electrolyte derangements themselves [7].

In critically ill patients, potassium abnormalities frequently coexist with acid-base disturbances, hypoxia, and ischemia, compounding their arrhythmogenic potential [8]. Evidence from observational studies has shown that both hypokalemia and hyperkalemia are associated with increased in-hospital mortality, prolonged hospital stay, and a higher likelihood of cardiac arrest [9]. Importantly, the risk of adverse outcomes is not confined to extreme derangements; even mild abnormalities outside the normal range can destabilize vulnerable myocardium. Emergency physicians and cardiologists are therefore tasked with rapid identification of potassium disturbances through bedside monitoring, electrocardiographic assessment, and prompt laboratory testing [10]. The electrocardiogram (ECG) serves as a vital diagnostic tool, as specific changes correlate with the degree of potassium abnormality. Hypokalemia typically produces ST-segment depression, T-wave flattening, and prominent U waves, while hyperkalemia may manifest with peaked T waves, widened QRS complexes, and eventual sine-wave morphology [11].

However, reliance solely on ECG changes is insufficient, as these may be absent or nonspecific, especially in chronic derangements. This underscores the importance of integrating clinical, laboratory, and electrocardiographic data in guiding urgent management [12].The clinical imperative in emergency care is not only to correct serum potassium levels but also to anticipate and prevent the arrhythmic consequences of these abnormalities. Treatment strategies range from intravenous potassium supplementation in hypokalemia to calcium gluconate, insulin-glucose infusion, β2-agonists, and renal replacement therapy in hyperkalemia. Given the narrow therapeutic window of potassium homeostasis, overcorrection itself can trigger dangerous rhythm disturbances, making continuous monitoring essential [13].

Aim

To evaluate the prevalence and clinical impact of serum potassium derangements in patients presenting with acute cardiac complaints to the emergency department.

Objectives

To determine the frequency of hypokalemia, normokalemia, and hyperkalemia among emergency cardiac care patients and to assess the association between the type of serum potassium abnormality and the corresponding type of arrhythmia (atrial, ventricular, or bradyarrhythmic/conduction).

## Materials and methods

This was a cross-sectional analytical study conducted at the Department of Emergency Cardiac Care, Shaikh Zayed Hospital, Lahore, Pakistan, from July 2024 till December 2024 (approval number: 1263/24). A total of 185 patients presenting with acute cardiac complaints were enrolled. A non-probability consecutive sampling technique was employed to recruit participants. Based on prior regional studies, the expected prevalence of dyskalemias among cardiac emergency patients was assumed to be approximately 45%, with a 95% confidence level and a 5% margin of error. This yielded a minimum required sample size of ≈190 participants. Additionally, considering an anticipated odds ratio (OR) of 2.0 for the association between potassium derangement and arrhythmias (based on prior observational studies), a sample size of around 180-190 patients provided a power of 80% to detect statistically significant differences between groups. The use of non-probability consecutive sampling introduces the potential for selection bias, as patients presenting during the study period may not fully represent the broader population of emergency cardiac patients. Individuals with less severe or atypical presentations might have been underrepresented, potentially influencing the observed frequency of dyskalemias and arrhythmias.

Inclusion criteria

Adult patients aged 18 years and above were eligible for inclusion if they presented with acute cardiac complaints such as chest pain, palpitations, syncope, presyncope, or dyspnea. Only patients with available serum potassium levels at the time of presentation and those who underwent continuous electrocardiographic (ECG) monitoring during their emergency evaluation were included in the study. This selection criterion ensured that all patients had the necessary laboratory and diagnostic data required to assess potassium derangements and the associated arrhythmias.

Exclusion criteria

Patients were excluded from the study if they had a history of chronic potassium supplementation, if their blood samples were hemolyzed, or if they had known congenital channelopathies, such as long QT syndrome or Brugada syndrome, which might confound the results. Additionally, patients with prior pacemaker implantation, incomplete medical records, or missing critical data were excluded to ensure the completeness and accuracy of the data set.

Data collection

At the time of presentation, venous blood samples were collected to measure serum potassium levels. Potassium derangements were classified as hypokalemia (<3.5 mmol/L), normokalemia (3.5-5.0 mmol/L), and hyperkalemia (>5.0 mmol/L). Simultaneously, 12-lead ECGs were performed and patients were placed on continuous cardiac monitoring. All arrhythmic events were documented and categorized as atrial (atrial fibrillation/flutter, supraventricular tachycardia), ventricular (ventricular tachycardia, fibrillation, premature ventricular contractions), or conduction abnormalities (bradyarrhythmia, AV block, bundle branch block). All arrhythmic events, including atrial arrhythmias (e.g., atrial fibrillation, atrial flutter, supraventricular tachycardia), ventricular arrhythmias (e.g., ventricular tachycardia, ventricular fibrillation, premature ventricular contractions), and conduction abnormalities (e.g., bradyarrhythmias, AV block, bundle branch block), were noted.

Statistical analysis

Data were entered and analyzed using SPSS version 26 (International Business Machines Corporation (IBM), Armonk, New York, USA). Descriptive statistics were applied to summarize baseline demographic and clinical characteristics. Chi-square test was used to compare categorical variables, while independent t-test or ANOVA was applied for continuous variables. Logistic regression analysis was performed to evaluate the association between potassium derangements and occurrence of arrhythmias, adjusting for potential confounders. A p-value of <0.05 was considered statistically significant.

## Results

A total of 185 patients were included, with a mean age of 58.6 ± 12.4 years. There were 112 males (60.5%) and 73 females (39.5%). The most common comorbidity was hypertension in 92 patients (49.7%), followed by diabetes mellitus in 64 (34.6%), ischemic heart disease in 58 (31.4%), chronic kidney disease in 41 (22.2%), and heart failure in 29 (15.7%). Chest pain was the predominant presenting complaint in 81 patients (43.8%), followed by palpitations in 44 (23.8%), dyspnea in 33 (17.8%), and syncope or presyncope in 27 (14.6%). Serum potassium assessment showed that 58 patients (31.4%) had hypokalemia, 92 (49.7%) were normokalemic, and 35 (18.9%) had hyperkalemia (Table 1).

**Table 1:** Baseline Demographic and Clinical Characteristics of Patients (N=185)

Variable	Value
Age, years, mean ± SD	58.6 ± 12.4
Gender, n (%)	
Male	112 (60.5)
Female	73 (39.5)
Hypertension, n (%)	92 (49.7)
Diabetes mellitus, n (%)	64 (34.6)
Chronic kidney disease, n (%)	41 (22.2)
Ischemic heart disease, n (%)	58 (31.4)
Heart failure, n (%)	29 (15.7)
Serum potassium category	
Hypokalemia (<3.5 mmol/L)	58 (31.4) mean ± SD: 3.12 ± 0.26 mmol/L
Normokalemia (3.5-5.0 mmol/L)	92 (49.7) mean ± SD: 4.26 ± 0.41 mmol/L
Hyperkalemia (>5.0 mmol/L)	35 (18.9) mean ± SD: 5.62 ± 0.48 mmol/L
Overall serum potassium (mean ± SD)	4.16 ± 0.82 mmol/L
Clinical presentation	
Chest pain	81 (43.8)
Palpitations	44 (23.8)
Syncope/presyncope	27 (14.6)
Dyspnea/heart failure	33 (17.8)

Hypokalemia was associated with the highest frequency of arrhythmias, as 39 patients (67.2%) experienced at least one arrhythmic event. Ventricular arrhythmias occurred in 21 patients (36.2%) with hypokalemia compared to 10 patients (10.9%) with normokalemia and six patients (17.1%) with hyperkalemia, a statistically significant difference. Hyperkalemia showed a stronger association with bradyarrhythmias and conduction blocks, which were observed in nine patients (25.7%) compared to four patients (6.9%) in hypokalemia and five patients (5.4%) in normokalemia. Atrial arrhythmias were also more common in hypokalemia, affecting 14 patients (24.1%) compared with 11 patients (12.0%) in normokalemia and three patients (8.6%) in hyperkalemia. Overall, arrhythmias occurred in 14 patients (40.0%) with hyperkalemia and 26 patients (28.3%) with normokalemia (Table 2).

**Table 2:** Association of Serum Potassium Levels With Arrhythmic Events (N=185) *p < 0.05. AF: atrial fibrillation, SVT: supraventricular tachycardia, VT: ventricular tachycardia, VF: ventricular fibrillation, PVCs: premature ventricular contractions.

Arrhythmia Type	Hypokalemia (n=58)	Normokalemia (n=92)	Hyperkalemia (n=35)	p-value	Chi-square value
Atrial arrhythmias (AF/flutter, SVT)	14 (24.1%)	11 (12.0%)	3 (8.6%)	0.04*	6.76
Ventricular arrhythmias (VT, VF, PVCs)	21 (36.2%)	10 (10.9%)	6 (17.1%)	<0.001*	14.52
Bradyarrhythmias/Conduction blocks	4 (6.9%)	5 (5.4%)	9 (25.7%)	0.002*	11.78
Any arrhythmia	39 (67.2%)	26 (28.3%)	14 (40.0%)	<0.001*	19.11

Multivariate logistic regression showed that hypokalemia significantly increased the odds of developing arrhythmias, with an odds ratio of 4.89 (95% CI: 2.45-9.75). Chronic kidney disease was also found to be an independent predictor of arrhythmias, with an odds ratio of 2.11 (95% CI: 1.04-4.27). Hyperkalemia increased the risk with an odds ratio of 1.75, but this was not statistically significant (Table 3).

**Table 3:** Logistic Regression Analysis of Potassium Derangements and Risk of Arrhythmias (N=185) *p < 0.05. Multivariate logistic regression was adjusted for potential confounders including age, gender, ischemic heart disease (IHD), diabetes mellitus (DM), and heart failure (HF) in addition to serum potassium categories and chronic kidney disease (CKD). Normokalemia served as the reference group for potassium category comparisons.

Variable	Odds Ratio (OR)	95% CI	p-value
Hypokalemia vs. Normokalemia	4.89	2.45-9.75	<0.001*
Hyperkalemia vs. Normokalemia	1.75	0.82-3.72	0.14
Age > 60 years	1.62	0.88-2.98	0.12
Male gender	1.09	0.62-1.91	0.76
Chronic kidney disease	2.11	1.04-4.27	0.03*

## Discussion

This study highlights the significant association between serum potassium derangements and the occurrence of arrhythmias in patients presenting to emergency cardiac care. Among 185 patients evaluated, dyskalemias were present in 50.3% of patients and arrhythmias were documented in 42.7% patients, with a markedly higher prevalence in those with potassium abnormalities compared to normokalemic individuals. Nearly one-third patients presented with hypokalemia, while almost one-fifth had hyperkalemia. Hypokalemia was strongly linked to ventricular arrhythmias, whereas hyperkalemia more commonly manifested as bradyarrhythmias and conduction blocks. The observed relationship between hypokalemia and ventricular arrhythmias is consistent with established electrophysiological principles. Potassium depletion prolongs repolarization and increases myocardial excitability, creating a substrate for early afterdepolarizations and reentrant circuits. This can explain the higher frequency of ventricular tachycardia, fibrillation, and ectopic beats observed in hypokalemic patients in our study. Previous research has similarly demonstrated that hypokalemia predisposes to torsades de pointes and sudden cardiac death, particularly in patients with structural heart disease or those receiving QT-prolonging medications. Our findings strengthen the clinical relevance of monitoring and correcting potassium in acute cardiac settings [14,15].

Hyperkalemia, in contrast, was more strongly associated with bradyarrhythmias and conduction disturbances. This aligns with the known mechanism whereby elevated extracellular potassium reduces the resting membrane potential, leading to slowed impulse conduction, QRS widening, and eventual conduction block. Severe hyperkalemia may culminate in sine-wave morphology and asystole, making it a true medical emergency. The finding that 25.7% of hyperkalemic patients in our cohort developed conduction abnormalities underscores the need for rapid recognition and intervention. Previous studies have also emphasized that even mild hyperkalemia significantly increases arrhythmic risk in patients with renal impairment, diabetes, or heart failure, populations that were well represented in our study [16]. Another notable observation was that atrial arrhythmias, including atrial fibrillation and supraventricular tachycardia, were more common in hypokalemic patients compared to normokalemic and hyperkalemic groups. This suggests that low potassium may not only predispose to ventricular excitability but also destabilize atrial conduction. This finding mirrors prior reports linking diuretic-induced hypokalemia to atrial fibrillation, reinforcing the importance of electrolyte surveillance in patients on long-term antihypertensive or diuretic therapy [17]. The clinical implications of these findings are considerable. Potassium derangements are frequently encountered in emergency cardiac care, particularly in patients with acute coronary syndromes, decompensated heart failure, or renal dysfunction. Our results support the routine assessment of serum potassium at presentation and the use of continuous ECG monitoring to detect early arrhythmic changes. The data also highlight that both hypo- and hyperkalemia are dangerous, although they predispose to different arrhythmic phenotypes. This distinction is important for guiding rapid, targeted interventions, such as potassium supplementation in hypokalemia and calcium or insulin-based therapies in hyperkalemia.

Our study adds to the growing evidence that even modest potassium abnormalities outside the normal reference range should not be overlooked in emergency settings. Early correction and prevention of overcorrection are equally critical, given that both extremes of potassium disturbance increase arrhythmic risk. For resource-limited settings, this emphasizes the need for rapid bedside testing, availability of intravenous therapies, and protocols to expedite management.

Limitations of this study must be acknowledged. The cross-sectional design precludes causal inference between potassium derangements and arrhythmic outcomes. The use of non-probability consecutive sampling introduces a potential selection bias, as the study population consisted only of patients presenting to a single tertiary emergency cardiac center during the study period. This approach may overrepresent individuals with more severe symptoms or advanced comorbidities, thereby limiting the generalizability of findings to the broader cardiac patient population or community settings. Additionally, arrhythmias were recorded only during the emergency department stay, possibly underestimating delayed or transient events. Serum potassium was measured only once at admission, introducing the potential for information bias, as it may not fully reflect the temporal fluctuation or peak severity of dyskalemia.

## Conclusions

Serum potassium derangements are strongly associated with arrhythmic events in patients. This study found that more than half of patients presenting to the emergency cardiac care unit had dyskalemias, with the majority leading to arrhythmias. Therefore, early testing for serum potassium levels, and its subsequent treatment, can not only prevent arrhythmias but also lead to early treatment and reversal of arrhythmia. Hypokalemia significantly increases the risk of ventricular and atrial arrhythmias, while hyperkalemia is more often linked to bradyarrhythmias and conduction blocks. Chronic kidney disease is an independent predictor of arrhythmias. These findings underscore the importance of early identification, continuous ECG monitoring, and prompt correction of potassium abnormalities in emergency settings.
